# A rare intraperitoneal inguinal bladder hernia (IBH) in a 58-year-old Indonesian male: A case report and review of the literature

**DOI:** 10.1016/j.ijscr.2023.108446

**Published:** 2023-07-01

**Authors:** Natalia Maria Christina, Christiano Tansol, Valeska Siulinda Candrawinata, Elrich Manggala Haryanto, Michaela Kemuning

**Affiliations:** a Department of Surgery, Faculty of Medicine, Pelita Harapan University, Siloam General Hospital, Tangerang, Indonesia; b Department of Urology, Faculty of Medicine, Pelita Harapan University, Siloam General Hospital, Tangerang, Indonesia; c Department of Surgery, Faculty of Medicine, Pelita Harapan University, Tangerang, Indonesia; d Faculty of Medicine, Pelita Harapan University, Tangerang, Indonesia

**Keywords:** Inguinal bladder hernia, Inguinal hernia, Herniorrhaphy, Open hernia repair surgery

## Abstract

**Introduction and importance:**

Inguinal bladder hernia (IBH) accounts for <5 % of inguinal hernias. As to our knowledge, this is the first case report of a rare intraperitoneal IBH in Indonesia.

**Case report:**

Here we present a case report of a 58-year-old Indonesian male complaining of a groin mass on the right side since 1 year ago, accompanied by lower urinary tract symptoms (LUTS), two-stage micturition, lower abdominal discomfort and pain during urinating and coughing. Ultrasound revealed widened inguinal canal containing peritoneum and “teardrop” lesion at the inguinal continuing until the right scrotal. The patient was scheduled for open repair of inguinal hernia (herniorrhaphy) with tension-free mesh. Intraoperative findings include the entire bladder herniation located at intraperitoneal.

**Clinical discussion:**

Symptoms of IBH include inguinal or scrotal swelling with or without pain, LUTS, two-stage micturition, to various symptoms owing to complications. Pre-operative imaging might help to confirm diagnosis. The definitive treatment of IBH is either reduction or resection of the herniated bladder followed by surgical repair (herniorrhaphy).

**Conclusion:**

IBH is rare but should be suspected in older males (≥50 years old), individuals with weak abdomino-pelvic musculature, and obesity. Pathologies of the bladder, such as bladder outlet obstruction (BOO), chronically distended bladder, and decreased bladder tone related to benign prostate hyperplasia (BPH) or bladder neck stricture can also increase risk of IBH. Treatment with open repair of inguinal hernia (herniorrhaphy) with tension-free mesh is the most common and preferred surgical approach.

## Highlights

• Inguinal hernia containing herniation of the bladder accounts for less than 5% of inguinal hernias.
• Male gender, age ≥50 years old, chronic urinary obstruction, weak pelvic musculature and obesity are risk factors of IBH
• Clinical presentation of IBH ranges from asymptomatic, symptoms related to inguinal or urinary tract to complications.
• Modalities such as ultrasonography (US), CT scan, and voiding cystourethrography have been useful in supporting diagnosis.
• Definitive treatment of IBH is surgical repair, with open surgical repair as the most common and preferred surgical approach.

## 1 Introduction

Inguinal hernia containing herniation of the bladder accounts for <5 % of inguinal hernias. Inguinal bladder hernia (IBH) could contain any part of the bladder (diverticulum, part of bladder, ureter, or entire bladder) [1,2]. Furthermore, a herniated bladder that is located in inguinoscrotal sac or also referred to as scrotal cystocele was first described in the literature by Levine in 1951 [3].

Several risk factors of IBH, including male gender, older age (≥50 years old), chronic urinary obstruction, weak pelvic musculature, and obesity [4], are known to increase its incidence as high as 10 % among obese men older than 50 years of age [5]. Clinical presentation of IBH ranges from asymptomatic to profound symptoms [3]. Symptoms of IBH include inguinal pain or swelling, lower urinary tract symptoms (LUTS), two-stage micturition, to various symptoms caused by its complications [1,[4], [5], [6]].

Pre-operative diagnosis of IBH is challenging as its symptoms are often not specific. However, modalities such as ultrasonography (US), cystoscopy, computed tomography (CT) scan, magnetic resonance imaging (MRI), and voiding cystourethrography have been useful in excluding other differential diagnosis [1,7]. Definitive treatment of IBH is surgical repair, with open surgical repair as the most common and preferred surgical approach [[6], [7], [8]].

This case report was conducted in line with the SCARE 2020 guideline: updating consensus Surgical Case Report (SCARE) guidelines [9]. As to our knowledge, this is the first case report of IBH in Indonesia. Here we present a 58-year-old Indonesian male with uncomplicated right-sided IBH treated with open surgical repair (herniorrhaphy) using the Lichtenstein technique and tension-free mesh.

## 2 Case report

A 58-year-old Indonesian male came to the surgery outpatient department complaining of a groin mass on the right side since 1 year ago. The mass gradually increased in size for the past year, beginning with the diameter of approximately 3 cm to 10 cm on presentation. The patient also complained of scrotal swelling since 8 months ago, measuring up to 12 cm on presentation.

He reported that pressure on the groin and scrotal swelling made him urinate more following spontaneous urination. He also observed that the mass decreased in size after urination. Other symptoms include lower abdominal discomfort and scrotal pain during urinating and coughing. Moreover, the patient also experienced urinary symptoms such as urinary incontinence, straining, increased frequency, and feeling of incomplete emptying. These symptoms had been present for a year and had become more and more severe. The patient denied any gastrointestinal symptoms (such as: nausea, vomiting, changes in bowel habit) or systemic symptoms (such as: fever, weight loss, fatigue).

Past medical history of the patient included diagnosis and surgery repair of inguinal hernia on the right side 6 years ago. There were no complications following the procedure. The patient reported no significant personal and family history of any disease, medication and/or genetic condition.

**Fig. 1:**
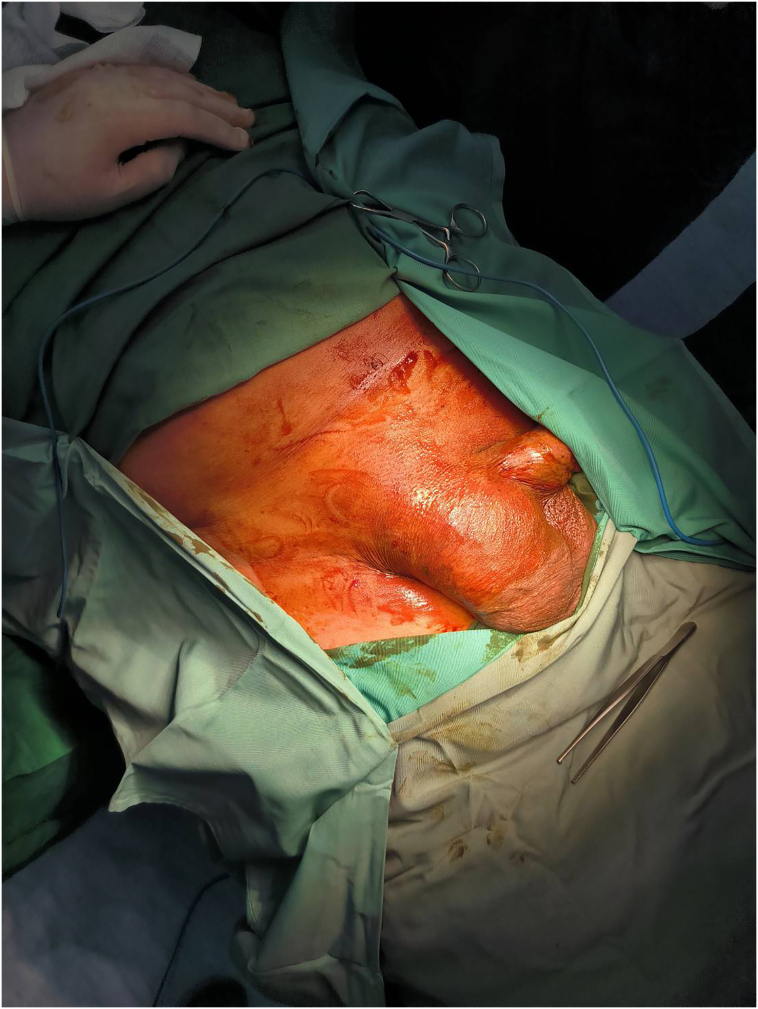
Pre-operative physical examination of the right inguinal region.

Physical examination—including vital signs—was within normal limits. The patient had normal BMI value of 23.6 kg/m^2^. Abdominal examination revealed pain on light and deep palpation at the hypogastric region. The patient graded the pain as 5 out of 10. Examination of the inguinal region revealed a 10 cm mass at the right inguinal with mild tenderness to palpation (Fig. 1). The mass was irreducible, with no discharge, no changes in skin colour nor temperature. There was also a noticeable right scrotal swelling, measuring approximately 12 cm, with mild pain on palpation, and no change in skin colour nor temperature. When pushed back, the patient reported feeling urgency to urinate. Moreover, the size of the mass increased when the patient was asked to do Valsalva manoeuvre.

**Fig. 2:**
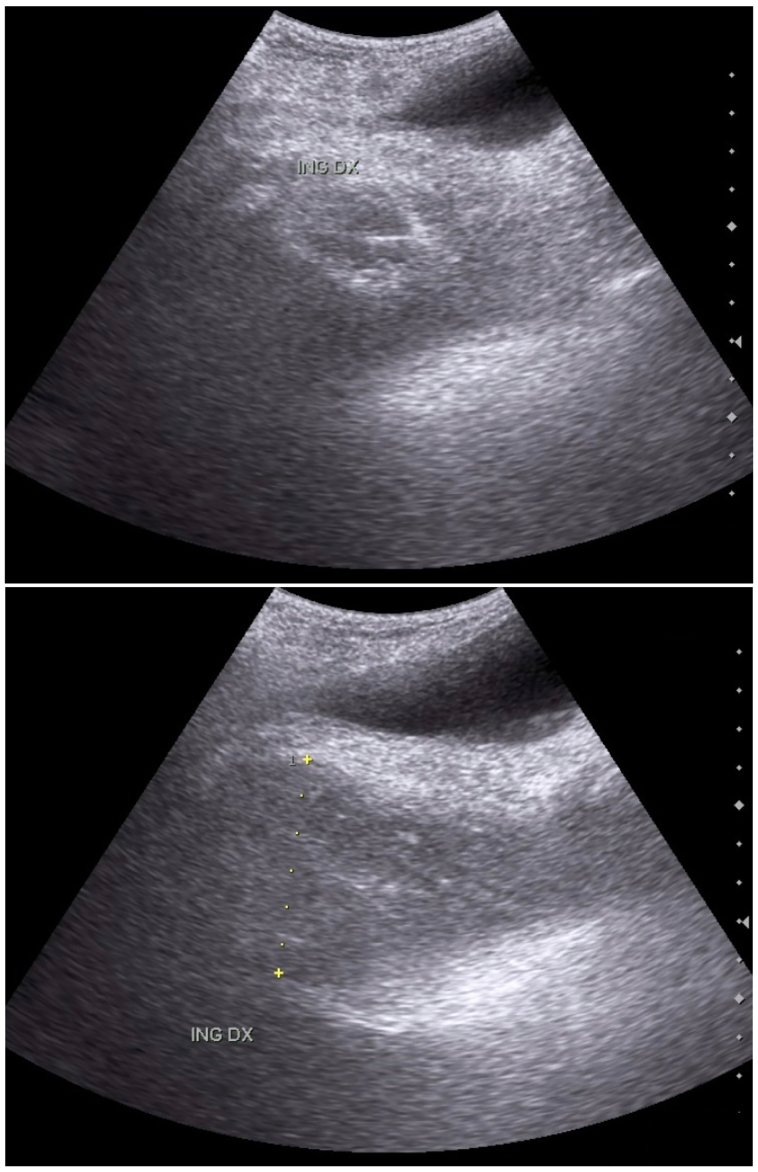
Abdominal and pelvis ultrasonography (US) revealed widened inguinal canal containing peritoneum and “teardrop” lesion at the inguinal continuing until the right scrotal.

Abdominal and pelvis ultrasonography (US) revealed widened inguinal canal containing peritoneum and “teardrop” lesion at the inguinal continuing until the right scrotal (Fig. 2). Other findings include fatty liver and normal prostate size (approximately 11.3 cc). Thickening of urinary bladder wall and stone of the urinary tract was not found.

Pre-operative laboratory examination was unremarkable. The patient was administered prophylaxis antibiotic: 2 g of Ceftriaxone via intravenous 1 h before incision. The patient immediately underwent surgery by an experienced general surgeon at a private general hospital. The surgical procedure was open repair of inguinal hernia (herniorrhaphy) with tension-free mesh. The surgery was done with the patient in supine position, under general anaesthesia.

**Fig. 3:**
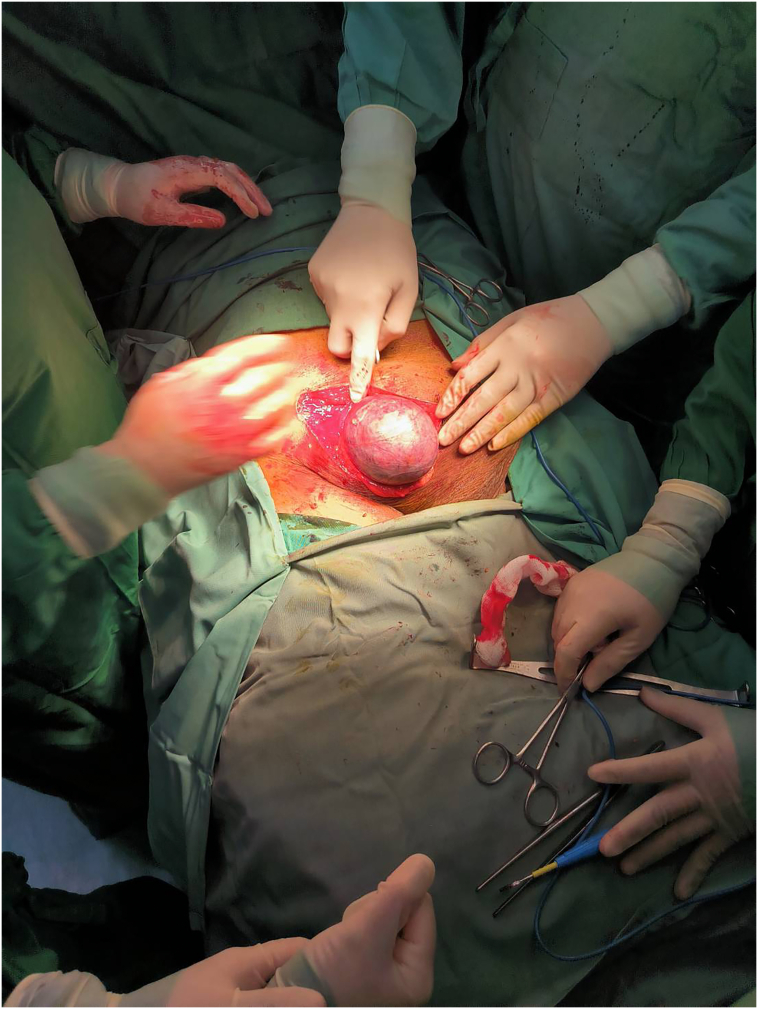
Intra-operative findings of bulging bladder when filled with Normal Saline via the Foley catheter, confirming the bladder herniation.

Skin incision was made superior of the inguinal ligament and layers are incised until hernia sac was found. Upon exploration, the hernia sac contained the entire urinary bladder covered with peritoneum (intraperitoneal). The bladder hernia was inguinoscrotal with approximated size of 12 cm × 8 cm × 10 cm. It was quite difficult to differentiate the hernia sac from the bladder due to its thickness. Therefore, the bladder was distended with Normal Saline via the Foley catheter and a large bulging was observed, confirming the bladder herniation (Fig. 3). The bladder was then reduced into the abdomen and the inguinal hernia and defect were repaired with a tension-free polypropylene mesh.

After the surgery, the patient stayed in the hospital for 1 day and complained of mild pain at excision area. At 1-day follow-up, the patient reported no symptoms of LUTS or signs of infection. The patient was discharged with medications: Cefixime 200 mg BID, Omeprazole 20 mg BID, Mefenamic acid 500 mg TID. Further follow-up was done at 3- and 7-days post-surgery at the outpatient department with absence of LUTS and adequate wound healing without any complications.

## 3 Discussion

Inguinal bladder hernia (IBH) contains part of the urinary bladder (diverticulum, part of bladder, ureter) or its entirity [1,2]. A herniated bladder that is located in inguinoscrotal sac or also referred to as scrotal cystocele was first described in the literature by Levine in 1951 [3]. IBH accounts for <5 % of inguinal hernias, with incidence as high as 10 % among obese men older than 50 years of age [1,2,5]. As to our knowledge, this is the first case report of IBH in Indonesia.

Majority of IBH occur in male with a 70 % to 95 % predominance [5,6]. Statistics have shown a ten-fold incidence increase of IBH in male compared to female [1]. Several identified risk factors are male gender, older age (≥50 years old), chronic urinary obstruction, weak abdomino-pelvic musculature, and obesity. These risk factors are related to several pathologies such as bladder outlet obstruction (BOO), chronically distended bladder, and decreased bladder tone [4,8]. BOO can be caused by benign prostate hyperplasia (BPH) or bladder neck stricture [7]. Most often, BOO occurs first, which then leads to bladder distension. In combination with weakening of abdominal and bladder wall the bladder slides through dilated inguinal ring [8].

Our patient had the risk factors of male gender, older than 50 years of age. Another possible factor contributing to IBH in this patient was history of right-sided hernia repair surgery, without mesh. Kim et al. described a patient who had herniorrhaphy 30 years before presentation, and a right scrotal mass was detected 5 years after the procedure [5]. Kraft et al. also described one of four patients who also had a previous herniorrhaphy several years before presentation [10]. However, the association between a history of herniorrhaphy and occurrence of bladder hernia is still uncertain. Direct, right-sided IBH is more common than left one which is also consistent with our case [1].

IBH mostly occur through the inguinal (75 %) and femoral (23 %) canals. The small remainder percentage occur though perineum, Linea alba (suprapubic), ischiorectal, obturator, rectus abdominus muscle (a Gironcoli hernia) and abdominal wall openings. Based on its anatomical relation with the parietal peritoneum, IBH can also be classified into paraperitoneal, intraperitoneal, and extraperitoneal, in accordance with its order of occurrence [11].

1. Paraperitoneal: the hernia sac may be direct or indirect and the extraperitoneal portion of the bladder lies along the medial wall of the sac.
2. Intraperitoneal: the bladder segment enters the peritoneum and is completely covered by it.
3. Extraperitoneal: the bladder herniates without any relationship to the peritoneum.

In our case, the herniated bladder was completely covered by the peritoneum, which can be classified as the intraperitoneal type.

**Figure:**
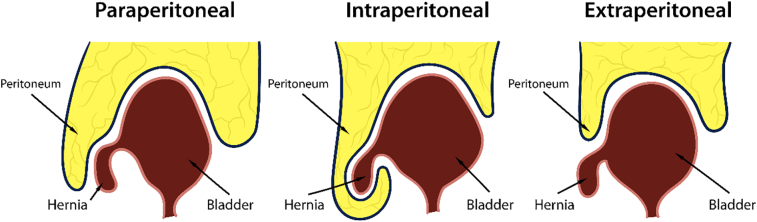

Clinical presentation of IBH ranges from asymptomatic to profound symptoms [1,5,6]. Asymptomatic patients or presentation of patient with unspecific symptoms poses a challenge in diagnosis of IBH resulting in <7 % of IBH diagnosed pre-operatively. However, in the last 10 years, this number has increased rapidly to 60 % as the result of incidental findings on more frequent imaging [4,6].

Symptoms of IBH include inguinal or scrotal swelling with or without pain, lower urinary tract symptoms (LUTS), two-stage micturition, to various symptoms as a result of its complications. Inguinal swelling is the most common symptom of IBH, found in approximately 60.3 % cases. In the beginning, inguinal swelling might be intermittent or affected by micturition (reduced after voiding) [[4], [5], [6]].

LUTS: voiding and storage symptoms are also common (present in 47.6 % cases) [[4], [5], [6]]. LUTS could be caused by BOO or complications such as urinary tract infection or cystolithiasis, but it is suspected to be mostly associated to IBH, as Kraft et al. described resolution of LUTS in four IBH patients with enlarged prostates [10]. Large IBH could present with two-stage micturition or “Mery's sign”, in which the first stage of micturition involves spontaneous voiding, and the second requires manual compression on the swelling [1,2].

The patient in this case report presented with a mildly painful inguinal swelling followed by a scrotal swelling, increasing in size up till 12 cm on presentation. The patient also experienced LUTS, including urinary incontinence, straining, increased frequency, and feeling of incomplete emptying. Report of two-stage micturition and decrease of mass size after urination also supports the diagnosis of IBH.

On physical examination, it might be difficult to exclude other differential diagnosis of bladder herniation, including herniated intestine or omentum, cord lipoma, testicular hydrocele or spermatocele [11]. Pre-operative imaging can be utilized in confirming diagnosis of IBH, such as ultrasonography (US), cystoscopy, computed tomography (CT) scan, magnetic resonance imaging (MRI), and voiding cystourethrography [4,6]. US is the first, most accessible and low-cost modality, with findings of a hypoechoic mass lesion from the bladder through the inguinal canal or the scrotum. It is also useful in detecting hydronephrosis or other disorders of the upper urinary tract. Cystoscopy is useful in assessing bladder outlet obstruction [4].

CT scan with or without contrast is the most frequent imaging, with findings of bladder protrusion indicating IBH, similar to findings in MRI. A “Mickey mouse sign” can also be found in presence of bilateral bladder hernia. Voiding cystourethrography is one of the best diagnostic imaging modalities in IBH, where a dumbbell-shaped bladder or a dog-ear shape of the bladder in the scrotum can be found [1,4]. In this patient, ultrasonography (US) was done pre-operatively, which revealed widened inguinal canal containing peritoneum and “teardrop” lesion at the inguinal continuing until the right scrotal, which supported the suspected diagnosis of IBH.

The definitive treatment of IBH is either reduction or resection of the herniated bladder followed by surgical repair (herniorrhaphy). Bladder reduction is preferred as it is associated with less complications, but bladder resection is recommended when there is bladder necrosis, a hernia neck with diameter < 0.5 cm, a true bladder diverticulum, or neoplasm in the herniated bladder [1,[5], [6], [7], [8]]. When bladder resection is performed, the vesicoureteral junction should be identified to minimize ureteral injury [5]. Catheterization before surgery is advised, especially when the diagnosis of IBH had been confirmed pre-operatively [4,6].

A systematic review by Branchu et al. found that open surgery was the most common surgical approach performed in 80.4 % of cases, with different techniques such as Lichtenstein (32.6 %), Bassini (15.2 %), Mac Way (4.3 %), Shouldice (2.2 %) [6]. A retrospective study published in 2001 in which 540 patients were treated with the Lichtenstein technique concluded that Lichtenstein's tension-free mesh hernia repair is a straightforward, safe, and efficient technique with a significantly low recurrence rate [1]. However, Khan et al. reported laparoscopic repair of bladder hernias which offered a shorter hospital stay, less analgesia requirement, quicker mobilization and return to daily activities, and improved cosmesis. The potential of laparoscopic surgery for bladder hernia repair should not be disregarded, but more studies concerning its recurrence and complications rate are required.

The surgical procedure done on the patient was open repair of inguinal hernia (herniorrhaphy) with tension-free mesh. Intra-operatively, a portion of the urinary bladder was found in the inguinal canal. It was quite difficult to differentiate the hernia sac from the bladder due to its thickness. Therefore, the bladder was distended with Normal Saline via the Foley catheter and a large bulging was observed, confirming the bladder herniation. The bladder was then reduced into the abdomen and the inguinal hernia and defect were repaired with a polypropylene mesh.

Complications related to surgical repair of inguinal hernia is damage of the bladder, but its rate has dropped as pre-operative diagnosis has increased significantly. Other possible complications might arise from bladder resection procedure, which could cause infection or decreased bladder capacity which would require subsequent treatment [5,8].

Findings in various follow up period between 3 days to 3 years included no reported recurrence was reported and improvement of LUTS after the surgical repair of hernia [6]. Limitation of this case report is a short period of follow-up (7 days), in which the patient did not show any signs and symptoms of complications or recurrence.

## 4 Conclusion

IBH is rare but should be suspected in older males (≥50 years old), individuals with chronic urinary obstruction, weak abdomino-pelvic musculature, and obesity. Pathologies of the bladder, such as bladder outlet obstruction (BOO), chronically distended bladder, and decreased bladder tone related to benign prostate hyperplasia (BPH), or bladder neck stricture can also increase risk of IBH. Treatment with open repair of inguinal hernia (herniorrhaphy) with tension-free mesh is the most common and preferred surgical approach.

## Patient perspective

At 1-day post-op follow up, the patient felt mild pain, but no symptoms of LUTS or signs of infection. Later on, at 3- and 7-days post op follow up, the patient reported no LUTS, minimal pain and expressed satisfaction in the scar result.

## Consent

Written informed consent was obtained from patient about these case reports writing and publishing. The patient understood well and gave consent. A copy of the written consent is available for review by the Editor-in-Chief of this journal on request.

## Ethical approval

As it is a case report, ethical approval is not required nor published by our institution.

## Funding

This research received no specific grant from any funding agency in the public, commercial, or not-for-profit sectors.

## Author contribution

NMC, VS: the conception and design of the study, or acquisition of data, or analysis and interpretation of data, drafting the article or revising it critically for important intellectual content, final approval of the version to be submitted; CT, EMH, MK: acquisition of data, drafting the article critically for important intellectual content, final approval of the version to be submitted.

## Guarantor

Natalia Maria Christina acts as the guarantor of this study.

## Research registration number

N/A.

## Declaration of competing interest

The authors declare that there is no conflict of interest.
